# Elucidating the association of obstructive sleep apnea with brain structure and cognitive performance

**DOI:** 10.1186/s12888-024-05789-x

**Published:** 2024-05-06

**Authors:** Jiahao Bao, Zhiyang Zhao, Shanmei Qin, Mengjia Cheng, Yiming Wang, Meng Li, Pingping Jia, Jinhui Li, Hongbo Yu

**Affiliations:** 1 Department of Oral and Cranio-Maxillofacial Surgery, Shanghai Ninth People’s Hospital, College of Stomatology, Shanghai Jiao Tong University School of Medicine, National Center for Stomatology, National Clinical Research Center for Oral Diseases, Shanghai Key Laboratory of Stomatology and Shanghai Research Institute of Stomatology, No. 639 Zhizaoju Road, Shanghai, China; 2 Department of Neurology, Nanjing Jinling Hospital, Affiliated Hospital of Medical School, Nanjing University, Nanjing, China; 3 JC School of Public Health and Primary Care, The Chinese University of Hong Kong, Shatin, Hong Kong; 4 Department of Urology, Stanford University Medical Center, Stanford, CA USA

**Keywords:** Obstructive sleep apnea, Brain Structure, Cognitive Performance, Genome-wide association study, Mendelian randomization, Hippocampus

## Abstract

**Background:**

Obstructive sleep apnea (OSA) is a pervasive, chronic sleep-related respiratory condition that causes brain structural alterations and cognitive impairments. However, the causal association of OSA with brain morphology and cognitive performance has not been determined.

**Methods:**

We conducted a two-sample bidirectional Mendelian randomization (MR) analysis to investigate the causal relationship between OSA and a range of neurocognitive characteristics, including brain cortical structure, brain subcortical structure, brain structural change across the lifespan, and cognitive performance. Summary-level GWAS data for OSA from the FinnGen consortium was used to identify genetically predicted OSA. Data regarding neurocognitive characteristics were obtained from published meta-analysis studies. Linkage disequilibrium score regression analysis was employed to reveal genetic correlations between OSA and related traits.

**Results:**

Our MR study provided evidence that OSA was found to significantly increase the volume of the hippocampus (IVW β (95% CI) = 158.997 (76.768 to 241.227), *P* = 1.51e-04), with no heterogeneity and pleiotropy detected. Nominally causal effects of OSA on brain structures, such as the thickness of the temporal pole with or without global weighted, amygdala structure change, and cerebellum white matter change covering lifespan, were observed. Bidirectional causal links were also detected between brain cortical structure, brain subcortical, cognitive performance, and OSA risk. LDSC regression analysis showed no significant correlation between OSA and hippocampus volume.

**Conclusions:**

Overall, we observed a positive association between genetically predicted OSA and hippocampus volume. These findings may provide new insights into the bidirectional links between OSA and neurocognitive features, including brain morphology and cognitive performance.

**Supplementary Information:**

The online version contains supplementary material available at 10.1186/s12888-024-05789-x.

## Background

Obstructive sleep apnea (OSA) is a widespread chronic sleep-related breathing disorder characterized by recurrent complete or partial collapses in the upper airway during sleep [1, 2]. It affects approximately 9% to 38% of the general adult population, and its prevalence steadily rises with increasing age [3]. Patients with OSA may suffer from insomnia, excessive daytime sleepiness, tiredness, inattention, or headaches due to frequent occurrences of blocked airways [4]. Intermittent hypoxia and sleep fragmentation are key features of OSA, triggering subsequent events such as oxidative stress, systemic inflammation, excessive activity of the sympathetic nervous system, metabolic imbalance, and hemodynamic swings [5, 6]. It is multifactorial and associated with many co-morbidities, including cardiovascular, metabolic and neurocognitive abnormalities [1, 7, 8]. Specifically, obesity or higher body mass index (BMI) are significantly associated with OSA, whereby obese patients are prone to developing OSA. Additionally, it has been reported that obesity is also a risk factor for exacerbating OSA, and developing OSA is also related to subsequent weight gain [9, 10]. During the past few decades, growing evidence elucidates the relationship between OSA and alterations of brain structure and cognitive performance. The brain might be influenced by oxidative tissue damage, apoptotic neuronal cell death, inflammation, and intracellular edema due to the presence of OSA [11, 12]. Also, brain neural injury contributes to cognitive performance change [13, 14]. However, the causal association of OSA with brain structure and cognitive performance remains unclear.

Magnetic resonance imaging (MRI) is a useful tool for examining brain structure and identifying structural alterations in individuals with OSA. Previous observational studies have reported that OSA is associated with cortical thickening or thinning in different regions, such as the rostral middle frontal lobe, frontal pole, postcentral, insula, and temporal pole [15–17]. It is reported that brain subcortical structure also changes in OSA patients. Kumar et al. found that the global putamen volume was significantly higher in the OSA patients, and M.Macey et al. demonstrated OSA-related brain change in hippocampal subfields [18, 19]. Lee et al. found that OSA status changes were significantly associated with white matter integrity and cognition [6]. These studies reached inconclusive findings about brain alterations in OSA patients, with some reporting cortical thinning and others finding no significant relationships or inverse associations [20]. It is still unclear whether OSA causes or results from these structural morphological changes. Figuring out the alteration of brain structure may provide insights into mechanisms of cognitive and behavioral changes observed in OSA patients. Moreover, traditional observational studies exhibit limitations such as small sample sizes, inconsistent results, existing confounding factors, and measurement errors. Given the increase in OSA prevalence with aging, environmental confounding, such as socioeconomic status, lifestyle habits, smoking, drinking, and obesity, might impede the ability of researchers to explore the causal association by traditional observational studies [10, 21]. It is difficult to make causal inferences based on these observational studies due to possible confounders and reverse causality. Therefore, further exploration is necessary to better understand the direction of these associations.

Mendelian randomization (MR) applies genetic variants as instrumental variables (IVs) of exposure to estimate the potential causal association between exposure and outcome [22, 23]. Because of the random allocation of alleles at conception, MR can avoid the influence of potential confounders to logically estimate the causal sequence [24, 25]. Previous studies have explored the relationship of OSA with cardiovascular disease, COVID-19, Alzheimer's disease, and Parkinson’s disease [26–28]. And the associations between structural alterations in the brains with sleep traits such as insomnia and sleep efficiency were identified. However, the underlying genetic and environmental factors associated with OSA and brain alterations remain poorly understood. The causal association of OSA with brain structure and cognitive performance requires further study. Based on summary-level genome-wide association study (GWAS) data for brain MRI measures and cognition-related phenotypes, this study applied the two-sample MR analysis to investigate the causal associations of OSA with brain structural morphology and cognitive performance. Additionally, genetic correlation, a population metric that describes the shared genetic architecture of various phenotypes, has not been described for OSA. Linkage disequilibrium score (LDSC) regression analysis is a reliable and efficient method to estimate the genetic correlation between different traits, which is based on summary GWAS data [29, 30]. We also applied LDSC regression analysis to reveal genetic correlations between OSA and relative traits in our study.

## Methods

### Study design

**Fig. 1:**
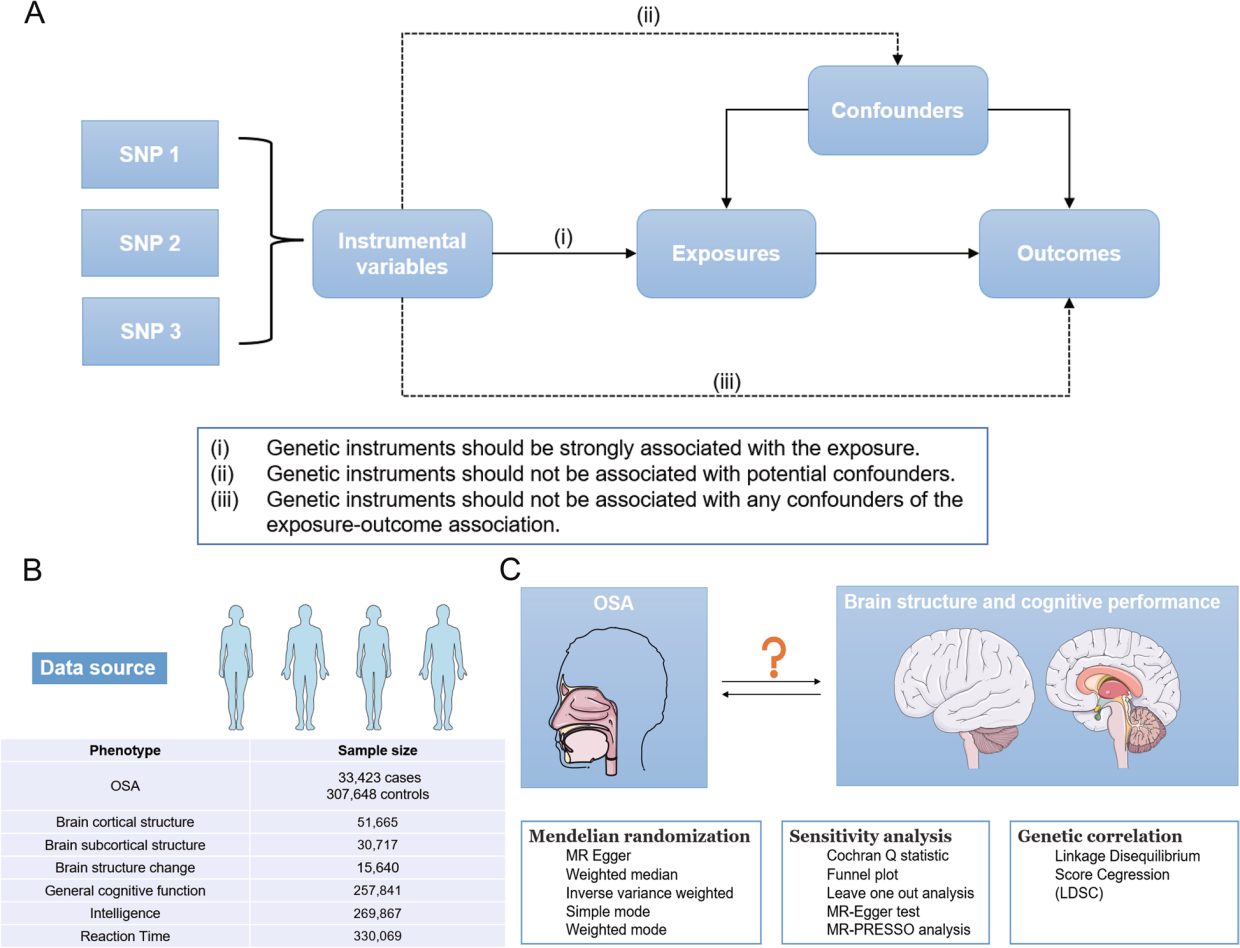
The overview of our study

The overview of the study is presented in Fig. 1. This study is reported according to the STROBE-MR (Strengthening the reporting of observational studies in epidemiology using mendelian randomization) guidelines (Additional file 1: STROBE-MR Checklist) and should rely on three assumptions [31, 32]. First, genetic instruments should be strongly associated with exposure. Second, genetic instruments should not be associated with potential confounders. Third, the genetic instruments should not be associated with any confounders of the exposure-outcome association.

### Data sources

A detailed description of the data sources is shown in Additional file 2: Table S1. The summary-level GWAS data for OSA were downloaded from the FinnGen consortium (Round 8), which contains 33,423 OSA cases and 307,648 controls [33, 34]. The diagnosis of OSA was made using the International Classification of Diseases codes (ICD-10: G47.3, ICD-9: 3472A), which were determined based on subjective symptoms, clinical examination, and sleep registration (AHI ≥ 5 events per hour or respiratory event index ≥ 5 events per hour).

The primary outcomes were as follows: brain cortical structure, brain subcortical structure, brain structure change across the lifespan and cognitive performance. The brain structure-related GWAS data were obtained from the Enhancing Neuro Image Genetics through Meta Analysis (ENIGMA) Consortium. We obtained the GWAS data of cortical thickness and surface area measures extracted from structure brain magnetic resonance images in 34 regions defined by the Desikan-Killiany atlas, which involved 51,665 individuals from 60 cohorts across the globe, primarily of European descent (~ 94%) [35]. For each specific region, data with global weighted suggested regional surface area or thickness were controlled for global measures (total surface area or average thickness). The brain subcortical structure-related GWAS of the intracranial volume (ICV) and the volumes of 7 subcortical regions (nucleus accumbens, amygdala, caudate, hippocampus, pallidum, putamen, and thalamus) corrected for the ICV, which derived from MRI scan of 30,717 individuals from 50 cohorts [36]. ICV, a measure of global brain size, was calculated as 1/(determinant of a rotation-translation matrix obtained after affine registration to a common study template and multiplied by the template volume (1,948,105 mm3)) [36]. The GWAS meta-analysis data for brain structure change across the lifespan was also obtained from the ENIGMA Consortium [37]. It comprised the 15 brain structures (total brain, surface area, cortical thickness, amygdala, caudate, cerebellar gray matter, cerebral and cerebellar white matter, cortical gray matter, hippocampus, lateral ventricles, nucleus accumbens, putamen, and thalamus) and the change rates were computed from longitudinal MRI data from 15,640 individuals covering the lifespan. As for cognitive performance, we downloaded summary statistics data for general cognitive function (*N* = 257,841) from the Social Science Genetic Association Consortium (SSGAC) [38], intelligence (*N* = 269,867) from Savage et al. [39] and reaction time (*N* = 330,069) from Davies et al. [40].

### Selection of instrumental variables

We extracted single nucleotide polymorphisms (SNPs) associated with OSA at the genome-wide level of significance threshold (*P* < 5e-8). In reverse MR analysis, we also extracted SNPs associated with each exposure at a genome-wide level of significance (*P* < 5e-6) except for brain subcortical structure, for which we relaxed the significance threshold to *P* < 5e-5 to include more IVs. Then, linkage disequilibrium (LD) clumping was utilized to select independent SNPs using the criteria of r^2 = 0.01 and the distance of 10,000 kb. To evaluate the weak instrument bias of the IVs, we calculated the F-statistic (F = beta^2/se^2) for each SNP and calculated a general F-statistic for all SNPs. SNP with an F-statistic less than 10 was considered as a low probability of a weak instrument bias and would be removed [41].

### Mendelian randomization analysis

Wald ratio was used to estimate the effect of exposure on the outcome for each SNP, and then we combined each SNP’s effect size using the inverse variance–weighted (IVW) method to obtain an overall estimate. Multiple methods, including IVW, MR-Egger regression, weighted median, weighted model, and simple mode, were applied to evaluate whether there was a causal association between exposure and outcome, in which IVW was considered as the major outcome [42, 43]. The weighted median method allows for the correct estimation of causal association when up to 50% of instrumental variables are invalid, whereas MR Egger allows all the instruments to be invalid, which makes it possible to evaluate the existence of pleiotropy with the intercept term [43, 44].

Then, the Cochrane’s Q value and the funnel plot were applied to detect the heterogeneity [45]. The MR-Egger intercept and MR Pleiotropy RESidual Sum and Outlier (MR-PRESSO) methods were applied to assess horizontal pleiotropy [46, 47]. Leave-one-out analysis was used to identify potential outliers which could cause strong bias in the result. The outliers would be removed, and MR analyses would be re-conducted. We then calculated the power of our MR analyses using an online MR power calculation tool (https://sb452.shinyapps.io/power/) provided by Stephen Burgess [48]. We also searched IVs in the website tool PhenoScanner V2 (www.phenoscanner.medschl.cam.ac.uk), a database of human genotype–phenotype associations, to check whether these SNPs were related to the potential phenotypes including obesity, body mass index (BMI), alcohol intake and smoking [49, 50]. IVs associated with these confounders significantly (*P* < 5e-8) were removed and MR analyses were re-conducted. Additionally, multivariate MR (MVMR) analysis was conducted in our study regarding the potential impact of obesity or BMI on OSA. The GWAS meta-analysis data for BMI was obtained from the GIANT Consortium [51]. MVMR was performed using the MVMR (version 0.4) package in R.

### Genetic correlation analysis

The genetic correlation between OSA and relative traits was evaluated using LDSC regression analysis [52]. European ancestry information from the 1000 Genomes Project was used as the reference for linkage disequilibrium, which was appropriate for the European GWAS project [53]. GWAS summary statistics were reformatted using munge_sumstats.py, and then LDSC regression analysis was conducted by ldsc.py according to the command line tool “ldsc” (https://github.com/bulik/ldsc).

### Statistical analysis

All statistical analyses were performed using the R package “TwoSampleMR”, “MR-PRESSO” and “MVMR” in R Software 4.1.2 [47, 54, 55]. Applying Bonferroni correction for multiple testing, a *P* value below 0.05/165 = 3.03e-04 was considered significant for MR analysis. Estimates with *P* below 0.05 but over 3.03E-04 were regarded as nominal significant, which still indicated a potential association. In LDSC regression analysis, a *P* value below 0.05/12 = 4.17e-03 was considered as significant after Bonferroni correction.

## Results

### The causal effect of OSA on brain structure and cognitive performance

**Fig. 2:**
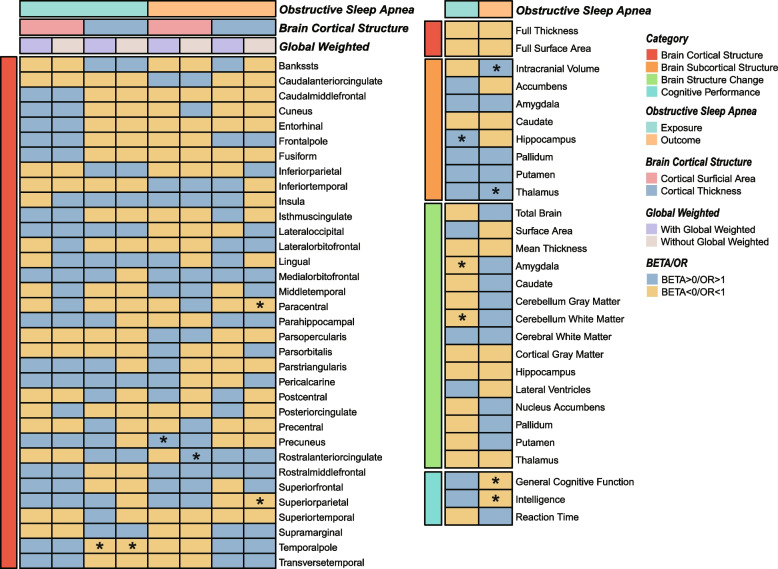
Heatmap shows the results of bidirectional MR analysis using IVW method to elucidate the association of obstructive sleep apnea with brain structure and cognitive performance. *P* value < 0.05 was marked as “*”

**Fig. 3:**
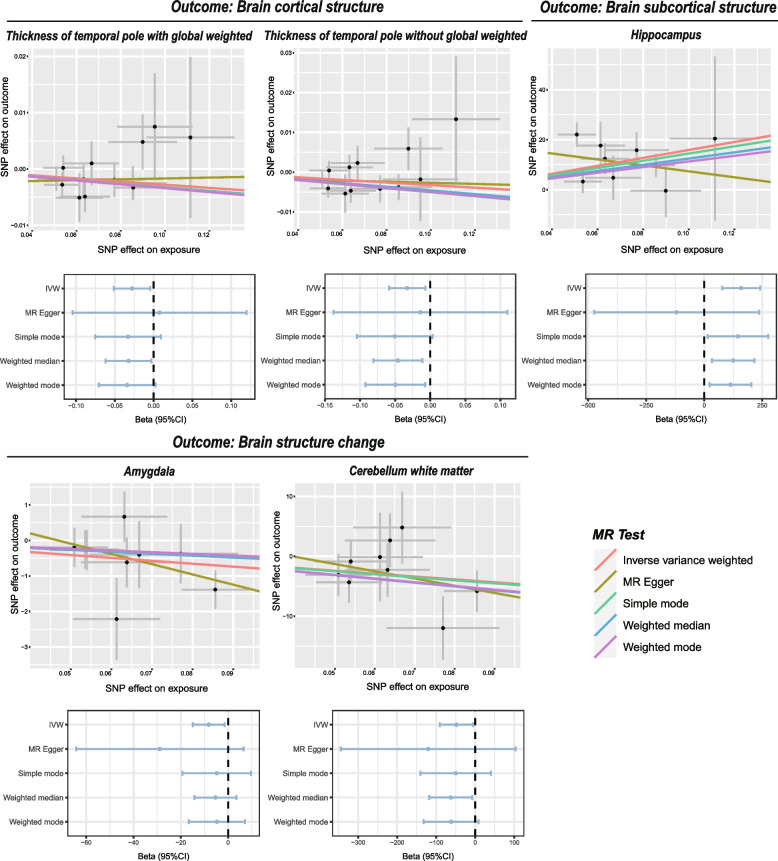
Scatter plots and forest plots show the causal association of OSA with brain structure and cognitive performance

The details of SNPs used as instrumental variables are displayed in Additional file 2: Table S2. In total, 13 SNPs (rs10507084, rs10986730, rs11075985, rs113955098, rs11981973, rs12511115, rs12788184, rs140896965, rs59333125, rs742760, rs76229479, rs78549563, rs9551988) were extracted to predict OSA genetically and the F statistics for each IV were all greater than the threshold 10, indicating that all IVs had sufficient validity. We performed a univariate MR analysis to explore the effect of OSA on brain cortical structure (including global surface area and thickness as well as 34 cortical regions with and without global weighted), brain subcortical structure (ICV and the volumes of 7 subcortical regions), brain structure change across the lifespan (total brain, surface area, cortical thickness, and other 12 brain regions) and cognitive performance (general cognitive function, intelligence and reaction time) (Figs. 2 and 3, Additional file 2: Tables S3 and S4). The causal effect of each SNP on brain structure was shown in Additional file 3: Figure S1. For brain cortical structure, OSA was found to decrease the thickness of the temporal pole with global weighted (IVW β (95% CI) = -0.028 (-0.051 to -0.005), *P* = 0.019) and without global weighted (IVW β (95% CI) = -0.033 (-0.058 to -0.007), *P* = 0.013) in a nominal significance level. For brain subcortical structure, OSA was found to significantly increase the volume of the hippocampus (IVW β (95% CI) = 158.997 (76.768 to 241.227), *P* = 1.51e-04). In addition, we observed OSA was nominally associated with the longitudinal change of amygdala (IVW β (95% CI) = -8.191 (-14.930 to -1.452), *P* = 0.017) and cerebellum white matter (IVW β (95% CI) = -48.260 (-91.034 to -5.486), *P* = 0.027) covering the lifespan. No causal effects of OSA on cognitive performance were found in our study. In sensitivity analyses, no heterogeneity was observed by Cochran Q statistic and funnel plots (Additional file 3: Figure S2, Additional file 2: Table S5). Leave-one-out analysis suggested that the results were not affected by a single outlying variant (Additional file 3: Figure S3). All *P*-values of MR-Egger intercept tests and the MR-PRESSO global tests were greater than 0.05, suggesting no horizontal pleiotropy existed in our MR analysis (Additional file 2: Table S6). Moreover, the results of MR-PRESSO analyses were consistent with the IVW method, and no outliers were identified (Additional file 2: Table S7).

Then, we searched 13 SNPs in PhenoScanner V2 and identified 6 SNPs were associated with potential confounders. rs10986730, rs11981973, rs11981973, rs9551988 and rs11981973 were associated with BMI and body fat. rs11075985 was associated with diabetes, vascular or heart problems, sleep duration, snoring, and some obesity-related phenotypes including BMI, body fat, body size, waist circumference, and waist- hip ratio. We removed these SNPs, re-conducted MR analysis, and found estimates were consistent with the previous findings. Also, OSA was found to increase the volume of hippocampus (IVW β = 138.87, se = 57.59, *P* = 0.0159), suggesting that the causal association between OSA on the volume of the hippocampus was not violated by potential confounders (Additional file 3: Figure S4). Meanwhile, MVMR analyses were conducted (Additional file 3: Table S8). After adjusting for BMI, OSA was found to increase the thickness of the temporal pole with global weighted (IVW β = 0.02, *P* = 0.03) or without global weighted (IVW β = 0.02, *P* = 0.056). The association between OSA and the volume of hippocampus was consistent before and after excluding the effect of potential confounders (BMI, body fat, body size, waist circumference, and waist-hip ratio, diabetes, vascular or heart problems, sleep duration, and snoring).

### The causal effect of neurocognitive features on OSA risk

We further performed the reverse two-sample MR analysis to explore whether there was a bidirectional link between neurocognitive features and OSA risk. The characteristics of IVs used in the reverse MR analysis were shown in Additional file 2: Table S2.

After Bonferroni correction, there was no significant causal effect of brain structure and cognitive performance on OSA (Additional file 3: Figure S5, Additional file 2: Tables S9 and S10). However, *P* < 0.05 was still considered indicative of evidence for a potential association. The results of the IVW estimate suggested brain cortical structure, including thickness of paracentral without global weighted (IVW OR (95% CI) = 0.297 (0.116 to 0.760), *P* = 0.011) and thickness of superior parietal without global weighted (IVW OR (95% CI) = 0.503 (0.255 to 0.991), *P* = 0.047) had protective effects on OSA. Cognitive performance, including general cognitive function (IVW OR (95% CI) = 0.824 (0.736 to 0.922), *P* = 0.0008) and intelligence (IVW OR (95% CI) = 0.890 (0.800 to 0.989), *P* = 0.031) were found to have the causal effects on the risk of OSA. The surface area of precuneus with global weighted (IVW OR (95% CI) = 1.0005 (1.0002 to 1.0007), *P* = 0.0015), the surface area of rostral anterior cingulate without global weighted (IVW OR (95% CI) = 1.0010 (1.0003 to 1.0017), *P* = 0.0057), ICV (IVW OR (95% CI) = 1.0000003 (1.0000 to 1.0000005), *P* = 0.030) and thalamus volume (IVW OR (95% CI) = 1.0001 (1.0000 to 1.0002), *P* = 0.0127) had slightly effects on genetically predicted risk of OSA. Significant heterogeneity was observed in our IVs for the surface area of precuneus with global weighted, surface area of rostral anterior cingulate without global weighted, thickness of paracentral without global weighted, thalamus, general cognitive function and intelligence by Cochran Q statistic and funnel plots (Additional file 3: Figure S6, Additional file 2: Table S11). Heterogeneity was acceptable since we applied the random effects IVW method [56]. The MR-Egger intercept did not provide evidence for horizontal pleiotropy (Additional file 2: Table S12). Leave-one-out analysis suggested that the results were not affected by a single outlying variant (Additional file 3: Figures S7-S9). However, MR-PRESSO analysis identified outliers for Thalamus (rs2188399) and Intelligence (rs10119967). The outlier-corrected analyses were consistent with the raw test after removing these outlier SNPs (Additional file 2: Table S13).

 Additional file 2: Table S14 showed the statistical power of MR analyses. There was good power for MR examining links from OSA to Hippocampus (100%), Amygdala (100%), and Cerebellum white matter (100%). There was moderate power for MR examining links from general cognitive function (30.4%) and intelligence (24.3%) to OSA.

### Genetic correlation analysis

**Fig. 4:**
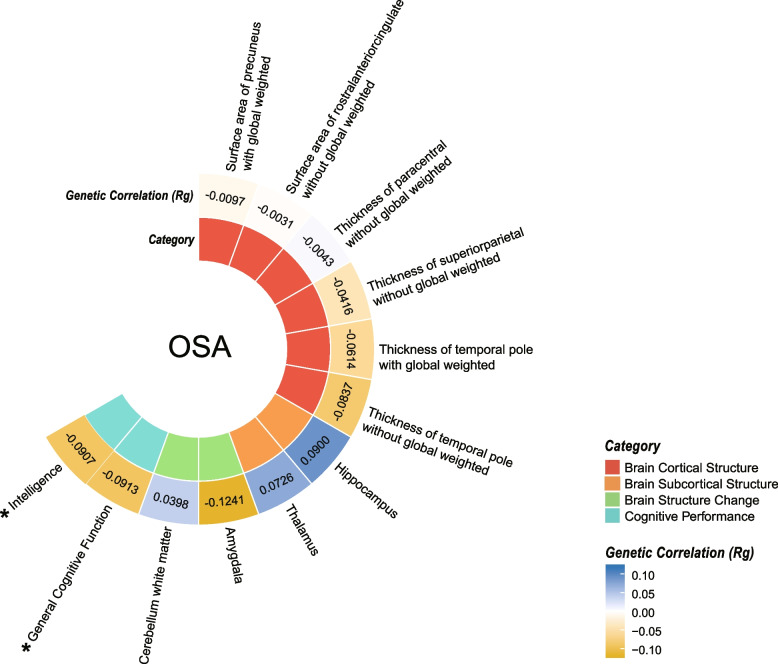
Genetic correlation analysis by the linkage disequilibrium score regression

Applying LDSC regression, we explored the genetic correlation of OSA with brain structure and cognitive performance, which were shown to have potential causal associations as described above (Fig. 4, Additional file 2: Table S15). There was evidence for genetic correlations of OSA with general cognitive function (Rg(se) = -0.0913 (0.026), *P* = 5.0e-4) and intelligence (Rg(se) = -0.0907 (0.0242), *P* = 2.0e-4) after Bonferroni correction. However, genetic correlation also suggested that OSA was not correlated with brain cortical structure (surface area of precuneus with global weighted, surface area of rostral anterior cingulate without global weighted, thickness of paracentral without global weighted, thickness of superior parietal without global weighted, thickness of temporal pole with and without global weighted), brain subcortical structure (hippocampus and thalamus), and brain structure change covering the lifespan (amygdala and cerebellum white matter).

## Discussion

To the best of our knowledge, this is the first large-scale MR study to comprehensively gain the inference about the causal association of OSA with brain structure and cognitive performance. In the present bidirectional MR study, we found that genetically predicted OSA was significantly associated with increased hippocampus volume adjusted for ICV. Nominally causal effects of OSA on brain structures, such as the thickness of the temporal pole with or without global weighted, amygdala structure change, and cerebellum white matter change, were observed across the lifespan. Bidirectional causal links were also detected between OSA and surface area of precuneus with global weighted, surface area of rostral anterior cingulate without global weighted, thickness of paracentral without global weighted, thickness of superior parietal without global weighted, ICV, thalamus volume, general cognitive function, intelligence and OSA. These MR findings could provide new insights into the bidirectional links of OSA with brain structural alterations and cognitive function.

A number of observational studies have investigated their association, applying neuroimaging tools and analytic methods such as MRI, voxel-based morphometry (VBM), and FreeSurfer [57]. However, observed phenomena varies substantially across different studies, and the findings are not always concordant among different neuroimaging studies; thereby, the impacts of OSA on brain subfields are controversial and not yet conclusive [58, 59]. In MR-based analysis, using the genetic variants associated with one of the traits as causal instruments, the correlations between OSA and brain alterations were revealed (Fig. 2). Genetic variants are randomly distributed during meiosis and fertilization, making them largely unaffected by self-selected behaviors, which avoid bias from confounding factors and reverse causality [60]. With regard to hippocampus volume, a part of the brain subcortical structure in the inferior part of the temporal lobe, we revealed OSA had a causal effect on increasing the volume of the hippocampus significantly (IVW β (95% CI) = 158.997 (76.768 to 241.227), *P* = 1.51e-04; Additional file 2: Table S4), while the genetic correlation was also positive but not significant. Herein, no heterogeneity and heterogeneity were detected (Additional file 2: Tables S5-S7). Moreover, to avoid potential confounding, we also re-conducted MR analysis after filter SNPs, which could be violated by potential confounders, including obesity, BMI, alcohol intake, and smoking, using Phenoscanner, and obtained consistent results, which yielded a robust estimate. It’s important to notice that GWAS data of nine subcortical structures utilized in our study were adjusted for total ICV, thereby genetic variants were independent of global head size. Our results support Rosenzweig’s, Martineau-Dussault’s, Macey’s, and Cross’s findings. Rosenzweig et al. observed significant increases in hippocampal volumes in OSA patients in the cross-sectional study [59]. Martineau-Dussault et al. studied 73 men and 47 women using MRI to extract total hippocampal volumes and they reported a positive correlation between AHI and bilateral hippocampal volumes in women, while OSA did not affect hippocampal volumes in men [61]. Macey’s group found OSA was mainly accompanied by hippocampal volume increases, but some subfields of volume decreased [19]. Also, Cross et al. retrospectively analyzed 83 middle-aged to older adults and identified an increased volume of hippocampus in OSA patients [16]. In line with our study, all of these studies applied the Freesurfer automated method to extract and calculate the hippocampal volumes and normalize hippocampal volumes to the total ICV. However, some studies suggested no difference between OSA and the control group [11, 62, 63]. In addition, the majority of previous clinical MRI studies showed hippocampal volume was markedly decreased in OSA patients compared with the control group therefore, impaired hippocampal function may be due to the reduction in white or gray matter or both [64–67]. For instance, based on VBM, a multimodal meta-analysis from Huang’s group found significant gray matter volume shrinkage of the hippocampus in OSA patients [66]. Another pathology finding showed that hippocampal atrophy and demyelination were related to the increasing severity of OSA [68]. One possible explanation for these inconsistent results could account for different image analysis tools and statistical thresholds. It has been reported that the whole-brain VBM method was less sensitive to detecting abnormalities in small subcortical structures, and its pre-processing and different thresholds would markedly influence the results [69, 70]. Conversely, the Freesurfer automated method was proven to be more effective. Besides, these clinical neuroimaging studies were limited by the small sample size, different inclusion criteria, lack of OSA-standardized neuropsychological tests, and potential confounders, including age, obesity, and mixed diseases. The clinical characteristics and experimental conditions varied between studies substantially, which could explain the large heterogeneity in these observational studies [71].

During the OSA, intermittent hypoxia was considered as the key pathological feature, and the hippocampus is especially vulnerable to hypoxia. It can lead to neuronal impairment and dysfunction, including neuronal death, neuroinflammation, intracellular edema, reactive gliosis, dendritic reorganization, and neuronal branching [11, 19, 72]. Considering that hypoxia has adverse impacts on neurobiological processes, it’s reasonable that OSA can directly impact brain structures. Previous studies demonstrated that hippocampal volume increases may arise from inflammation in the acute phase, altered neurogenesis, glial response to hypoxia, and intracellular edema [73, 74]. Rosenzweig et al. supposed the compensatory mechanism at the early stage of OSA resulted in enlargement of the hippocampus, thereby the abnormal enlargement always occurred in patients who were relatively young and with no obvious comorbidities [59]. Lee et al. suggested hypertrophy of the right subiculum in hippocampus is caused by OSA-related inflammation and intracellular edema [11]. Glial response, which is an early inflammatory response following brain injury characterized by the proliferation of microglia and astrocytes, was also approved to contribute to the hypertrophy change [75]. In addition, Martineau-Dussault et al. found the association between AHI and hippocampus volume disappeared using free-water corrected volume, illustrating the role of intracellular edema caused by OSA [61]. Nevertheless, the underlying mechanism of hippocampal volume increase remains unclear and warrants further studies to elucidate. It is essential to further explore whether OSA can lead to brain function change or neuropsychiatric diseases mediated by the alteration of the hippocampus could be expected.

Although only one estimate was still significant after the Bonferroni correction, other nominally significant estimates should also be treated carefully. For the thickness of the temporal pole, an essential part of episodic memory and language, our findings are in general agreement with previous neuroimaging studies. As previously suggested, oxygen desaturation was associated with cortical thinning of the bilateral temporal pole in adult OSA patients, and decreased thickness was linked to a poorer ability to encode new information [16]. For pediatric OSA patients, temporal cortical thinning was also discovered [76]. Our estimates suggested that OSA causally decreased the cortical thickness of the temporal pole both with and without global weighted (Additional file 2: Table S4). Thinning temporal pole has also been reported in attention-deficit/hyperactivity disorder [77], Parkinson’s Disease [78] and major depressive disorder [79]. However, whether OSA leads to these neuropsychiatric disorders by influencing temporal pole remains yet to be explored. Besides, we found OSA was causally related to change rates for cerebellum white matter (IVW β (95% CI) = -48.260 (-91.034 to -5.486), *P* = 0.027) and amygdala (IVW β (95% CI) = -8.191 (-14.930 to -1.452), *P* = 0.017) throughout the lifespan (Additional file 2: Table S4), which suggested that OSA might affect the process of brain development or aging.

In reverse MR analysis, the increase of thickness of the paracentral and superior parietal without global weighted was suggestively associated with a decreased risk of OSA (IVW OR (95% CI) = 0.503 (0.255 to 0.991), *P* = 0.047) with no heterogeneity, no pleiotropy, and high statistical power (Additional file 3: Figure S3, Additional file 2: Tables S9 and S10). These findings were consistent with previous reports, in which researchers found the average cortical thickness of the paracentral was lower in OSA patients [11, 80]. Since these studies always focused on how OSA affected brain structure, further studies are required to fully comprehend the complex association and mechanism between them. We also found higher general cognitive function and intelligence tend to be associated with a lower risk of OSA. Moreover, the negative correlation was detected by LDSC analysis. Contrastingly, previous findings mainly aimed to explore the impact of OSA on cognitive performance. For instance, Zhang et al. estimated associations between Polygenic Risk Scores for OSA and cognitive function in Hispanic/Latino adults, and found that PRS for OSA was not associated with cognitive outcomes [21]. A recent meta-analysis revealed neurocognitive deficits were evident in children with OSA [81]. Therefore, it is worthwhile to examine further the role of cognitive performance as an OSA marker.

Our study has several strengths. To the best of our knowledge, it’s the first study that has applied an MR analysis to investigate the causal association of OSA with brain structure and cognitive function. The use of MR design and the large sample size of GWAS datasets mitigate reverse causality and biases owing to confounding [60]. We comprehensively investigated the relationship between the brain and OSA by using multiple evaluation indicators. The GWAS datasets applied were derived from a European population and all studies had genomic control. Besides, multiple sensitivity analyses were conducted. Hence, the conclusions may not be influenced by population stratification and genomic inflation. Additionally, we applied LDSC to detect genetic correlation in order to provide a contrast to the MR analysis, which implied the results of observational studies might be biased by confounders (Fig. 4).

A few limitations should be noticed as well. First, since the enrolled patients were mainly Europeans, and ethnicity might impact craniofacial anatomy traits and obesity liability in OSA patients, the results cannot be generalized to other ancestries [82]. Second, although several methods were applied to detect pleiotropy including MR‐Egger intercept and MR‐PRESSO, it’s difficult to avoid bias caused by horizontal pleiotropy completely. When analyzing polygenic features, it’s challenging to satisfy the rigid set of assumptions required by MR analysis. We have made an effort to eliminate any SNPs known to be pleiotropic, but we still cannot ensure that this assumption has not been violated. Third, some of our MR analyses did not have enough power to detect causal effects. Especially in reverse MR traits, heterogeneity and pleiotropy were detected while the causal effects were close to 1. Besides, for the lack of significant IVs, the genome-wide significance threshold was relaxed to *P* < 5e-5 or *P* < 5e-6, which might lead to weak IVs. Since the sample size of GWAS was quite large, it would be acceptable generally. Last, the results of MR estimates and genetic correlation analysis were not exactly consistent. Genetic correlation analysis only tested the correlation between two traits employing SNPs with minor effects from the genome instead of utilizing a causal inference framework, which is similar to observational studies and might be biased by confounders [29, 83], it is possible that the results of the two analytical approaches were distinct. In the future, the underlying mechanisms that connect OSA to altered brain structures and functions need to be investigated to elucidate the biological rationale. Since the description of brain substructures utilized in our study was based on available neuroimaging GWAS datasets, further MR analysis is expected to verify the association between OSA and the volume of hippocampus subfields.

## Conclusions

It’s the first MR analysis that investigates the causal association between OSA and brain cortical structure, brain subcortical structure, brain structural change across the lifespan, and cognitive performance. Our study provided more evidence that there was a causal association between OSA and the increase of hippocampus volume. To determine the association between OSA and brain structure and function, additional research into the underlying mechanism is required.

### Supplementary Information

**Supplementary Material 1. **

**Supplementary Material 2. **

**Supplementary Material 3. **

## Data Availability

All the data utilized in the present study had been publicly available, and the source of the data had been described in the main text and shown in Additional file 2: Table S1. Code is available from the corresponding author (Hongbo Yu) by request.

## Abbreviations

OSA: Obstructive sleep apnea
MR: Mendelian randomization
GWAS: Genome-wide association study
LDSC: Linkage disequilibrium score
MRI: Magnetic resonance imaging
IVs: Instrumental variables
STROBE-MR: Strengthening the Reporting of Observational Studies in Epidemiology Using Mendelian Randomization
ENIGMA: Enhancing Neuro Image Genetics through Meta Analysis
ICV: Intracranial volume
SSGAC: Social Science Genetic Association Consortium
SNPs: Single nucleotide polymorphisms
LD: Linkage disequilibrium
IVW: Inverse variance–weighted
