# Supplementary material for: Elucidating the association of obstructive sleep apnea with brain structure and cognitive performance
Source: BMC Psychiatry. 2024 May 6;24:338. doi: 10.1186/s12888-024-05789-x (PMC11071327; doi:10.1186/s12888-024-05789-x)
Supplement: Supplementary file 1 — Supplementary Material 1. [file 12888_2024_5789_MOESM1_ESM.pdf]

Additional file 1: STROBE-MR Checklist.

Checklist of recommended items to address in reports of Mendelian randomization studies[1 2].

Item No.	Section	Checklist item	Page No.	Relevant text from manuscript
1	**TITLE and ABSTRACT**	Indicate Mendelian randomization (MR) as the study's design in the title and/or the abstract if that is a main purpose of the study	1-3	"We conducted a two-sample bidirectional Mendelian randomization (MR) analysis to investigate the causal relationship between OSA and a range of neurocognitive characteristics including brain cortical structure, brain subcortical structure, brain structural change across the lifespan, and cognitive performance."
	**INTRODUCTION**			
2	**Background**	Explain the scientific background and rationale for the reported study. What is the exposure? Is a potential causal relationship between exposure and outcome plausible? Justify why MR is a helpful method to address the study question	3-4	"During the past several decades, growing evidence elucidates the relationship between OSA and alterations of brain structure and cognitive performance."<br>"Figuring out the alteration of brain structure may provide insights into mechanisms of cognitive and behavioral changes observed in OSA patients, but it's still unknown whether these structural morphology alterations occur as a result or a cause of OSA. Moreover, these traditional observational studies exhibit limitations such as small sample size, inconsistent results, existing confounding factors and measurement errors. It's difficult to make causal inferences based on these observational studies due to reverse causality. Therefore, further exploration is necessary to better understand the direction of these associations."<br>"Previous studies have explored the relationship of OSA with cardiovascular disease, COVID-19, Alzheimer's disease and Parkinson's disease (26-28). However, the causal association of OSA with brain structure and cognitive performance has not been reported yet."
3	**Objectives**	State specific objectives clearly, including pre-specified causal hypotheses (if any). State that MR is a method that, under specific assumptions, intends to estimate causal effects	4	"Mendelian randomization (MR) applies genetic variants as instrumental variables (IVs) of exposure to estimate potential causal association between exposure and outcome (22,23)."<br>"In this study, based on summary-level genome-wide association study (GWAS) data for brain MRI measures and cognition-related phenotypes, we used two sample MR analysis to investigate the causal associations of

				OSA with brain structural morphology and cognitive performance.”
	**METHODS**			
4	**Study design and data sources**	Present key elements of the study design early in the article. Consider including a table listing sources of data for all phases of the study. For each data source contributing to the analysis, describe the following:		
		a) Setting: Describe the study design and the underlying population, if possible. Describe the setting, locations, and relevant dates, including periods of recruitment, exposure, follow-up, and data collection, when available.	5-6	“A detailed description of the data sources is shown in Additional File 2: Table S1.”<br>The summary-level GWAS data for OSA were downloaded from the FinnGen consortium (Round 8), which contains 33,423 OSA cases and 307,648 controls (33,34).<br>The primary outcomes were as follows: brain cortical structure, brain subcortical structure, brain structure change across the lifespan and cognitive performance.
		b) Participants: Give the eligibility criteria, and the sources and methods of selection of participants. Report the sample size, and whether any power or sample size calculations were carried out prior to the main analysis	5-6	“The diagnosis of OSA was made using the International Classification of Diseases codes (ICD-10: G47.3, ICD-9: 3472A), which were determined based on subjective symptoms, clinical examination, and sleep registration (AHI ≥ 5 events per hour or respiratory event index ≥ 5 events per hour).”<br>“The brain structure-related GWAS data were obtained from the Enhancing Neuro Image Genetics through Meta Analysis (ENIGMA) Consortium. We obtained the GWAS data of cortical thickness and surface area measures extracted from structure brain magnetic resonance images in 34 regions defined by the Desikan-Killiany atlas, which involved 51,665 individuals from 60 cohorts across the globe, primarily of European descent (~94%) (35).”<br>“The brain subcortical structure-related GWAS of the intracranial volume (ICV) and the volumes of 7 subcortical regions (nucleus accumbens…thalamus) corrected for the ICV, which derived from MRI scan of 30,717 individuals from 50 cohorts (36).”<br>“The GWAS meta-analysis data for brain structure change across the lifespan was also obtained from the ENIGMA Consortium (37). It comprised the 15 brain structures…”<br>“…we downloaded summary statistics data for general cognitive function (N=257,841) from Social Science

					Genetic Association Consortium (SSGAC) (38), intelligence (N= 269,867) from Savage et al. (39) and reaction time (N=330,069) from Davies et al.(40).”
		c)	Describe measurement, quality control and selection of genetic variants	6	We extracted single nucleotide polymorphisms (SNPs) associated with OSA at the genome-wide level of significance threshold (P < 5e-8). In reverse MR analysis, we also extracted SNPs associated with each exposure at a genome-wide level of significance (P < 5e-6) except for brain subcortical structure, for which we relaxed the significance threshold to P <5e-5 to include more IVs. Then, linkage disequilibrium (LD) clumping was utilized to select independent SNPs using the criteria of r^2 = 0.01 and the distance of 10,000 kb. To evaluate the weak instrument bias of the IVs, we calculated the F-statistic (F = beta^2/se^2) for each SNP and calculated a general F-statistic for all SNPs. SNP with F-statistic less than 10 was considered as a low probability of a weak instrument bias and would be removed (41).
		d)	For each exposure, outcome, and other relevant variables, describe methods of assessment and diagnostic criteria for diseases	5	“A detailed description of the data sources is shown in Additional File 2: Table S1.”<br>“The diagnosis of OSA was made using the International Classification of Diseases codes (ICD-10: G47.3, ICD-9: 3472A), which were determined based on subjective symptoms, clinical examination, and sleep registration (AHI ≥ 5 events per hour or respiratory event index ≥ 5 events per hour).”
		e)	Provide details of ethics committee approval and participant informed consent, if relevant	NA	NA
5	**Assumptions**		Explicitly state the three core IV assumptions for the main analysis (relevance, independence and exclusion restriction) as well assumptions for any additional or sensitivity analysis	5	“…should rely on three assumptions (31,32). First, genetic instruments should be strongly associated with exposure. Second, genetic instruments should not be associated with potential confounders. Third, the genetic instruments should not be associated with any confounders of the exposure-outcome association.”
6	**Statistical methods: main analysis**		Describe statistical methods and statistics used		
		a)	Describe how quantitative variables were handled in the analyses (i.e., scale, units, model)	7	“Wald ratio was used to estimate the effect of exposure on the outcome for each SNP and then we combined each SNP’s effect size using the inverse variance–weighted (IVW) method to obtain an overall estimate. Multiple methods including IVW, MR-Egger regression, weighted median, weighted model and simple mode

				were applied to evaluate whether there was a causal association between exposure and outcome, in which IVW was considered as the major outcome (42,43).”
	b)	Describe how genetic variants were handled in the analyses and, if applicable, how their weights were selected	6	“Selection of instrumental variables<br>We extracted single nucleotide polymorphisms (SNPs) associated with OSA at the genome-wide level of significance threshold (P < 5e-8). In reverse MR analysis, we also extracted SNPs associated with each exposure at a genome-wide level of significance (P < 5e-6) except for brain subcortical structure, for which we relaxed the significance threshold to P <5e-5 to include more IVs. Then, linkage disequilibrium (LD) clumping was utilized to select independent SNPs using the criteria of r^2 = 0.01 and the distance of 10,000 kb. To evaluate the weak instrument bias of the IVs, we calculated the F-statistic (F = beta^2/se^2) for each SNP and calculated a general F-statistic for all SNPs. SNP with F-statistic less than 10 was considered as a low probability of a weak instrument bias and would be removed (41).”
	c)	Describe the MR estimator (e.g. two-stage least squares, Wald ratio) and related statistics. Detail the included covariates and, in case of two-sample MR, whether the same covariate set was used for adjustment in the two samples	7	“…IVW was considered as the major outcome (42,43). The weighted median method allows for the correct estimation of causal association when up to 50% of instrumental variables are invalid, whereas MR Egger allows all the instruments to be invalid, which makes it possible to evaluate the existence of pleiotropy with the intercept term (43,44).”<br>“We also searched IVs in the website tool PhenoScanner V2 (www.phenoscanner.medschl.cam.ac.uk), a database of human genotype-phenotype associations, to check whether these SNPs were related to the potential phenotypes including obesity, body mass index (BMI), alcohol intake and smoking (49,50). IVs associated with these confounders significantly (P < 5E-8) were removed and MR analyses were re-conducted.”
	d)	Explain how missing data were addressed	NA	NA
	e)	If applicable, indicate how multiple testing was addressed	8	“Applying Bonferroni correction for multiple testing, a P value below 0.05/165=3.03e-04 was considered significant for MR analysis. Estimates with P below 0.05 but over 3.03E-04 were regarded as nominal significant, which still indicated a potential association.”

7	**Assessment of assumptions**	Describe any methods or prior knowledge used to assess the assumptions or justify their validity	7	"Then, the Cochrane's Q value and the funnel plot were applied to detect the heterogeneity (45). The MR-Egger intercept and MR Pleiotropy RESidual Sum and Outlier (MR-PRESSO) methods were applied to assess horizontal pleiotropy (46,47). Leave-one-out analysis was used to identify potential outliers which could cause strong bias to the result. The outliers would be removed, and MR analyses would be re-conducted."
8	**Sensitivity analyses and additional analyses**	Describe any sensitivity analyses or additional analyses performed (e.g. comparison of effect estimates from different approaches, independent replication, bias analytic techniques, validation of instruments, simulations)	7-8	"We also searched IVs in the website tool PhenoScanner V2 (www.phenoscanner.medschl.cam.ac.uk), a database of human genotype-phenotype associations, to check whether these SNPs were related to the potential phenotypes including obesity, body mass index (BMI), alcohol intake and smoking (49,50). IVs associated with these confounders significantly (P < 5E-8) were removed and MR analyses were re-conducted."<br>"The genetic correlation between OSA and relative traits was evaluated using LDSC regression analysis (51). European ancestry information from the 1000 Genomes Project was used as the reference for linkage disequilibrium, which was appropriate for the European GWAS project (52). GWAS summary statistics were reformatted using munge_sumstats.py and then LDSC regression analysis was conducted by ldsc.py according to the command line tool "ldsc" (https://github.com/bulik/ldsc)."
9	**Software and pre-registration**			
	a)	Name statistical software and package(s), including version and settings used	8	"All statistical analyses were performed using the R package "TwoSampleMR" and "MR-PRESSO" in R Software 4.1.2 (47,53)."
	b)	State whether the study protocol and details were pre-registered (as well as when and where)	5	"The study protocol was not pre-registered."
	**RESULTS**			
10	**Descriptive data**			
	a)	Report the numbers of individuals at each stage of included studies and reasons for exclusion. Consider use of a flow diagram	5-6	"… contains 33,423 OSA cases and 307,648 controls…<br>which involved 51,665 individuals from 60 cohorts across the globe, primarily of European descent (~94%) (35)… derived from MRI scan of 30,717 individuals from 50 cohorts (36)…. downloaded summary statistics data for general cognitive function (N=257,841) from

					Social Science Genetic Association Consortium (SSGAC) (38), intelligence (N= 269,867) from Savage et al. (39) and reaction time (N=330,069) from Davies et al.(40).”
		b)	Report summary statistics for phenotypic exposure(s), outcome(s), and other relevant variables (e.g. means, SDs, proportions)	NA	NA
		c)	If the data sources include meta-analyses of previous studies, provide the assessments of heterogeneity across these studies	NA	NA
		d)	For two-sample MR:<br>i. Provide justification of the similarity of the genetic variant-exposure associations between the exposure and outcome samples<br>ii. Provide information on the number of individuals who overlap between the exposure and outcome studies	5-6	“The summary-level GWAS data for OSA were downloaded from the FinnGen consortium (Round 8) …”<br>“…primarily of European descent (~94%) …”<br>“… was also obtained from the ENIGMA Consortium…”
11	**Main results**				
		a)	Report the associations between genetic variant and exposure, and between genetic variant and outcome, preferably on an interpretable scale	8	“The details of SNPs used as instrumental variables are displayed in Additional File 2: Table S2. In total, 13 SNPs were extracted to predict OSA genetically and the F statistics for each IV were all greater than the threshold 10, indicating that all IVs had sufficient validity.”<br>“The characteristics of IVs used in the reverse MR analysis were shown in Additional File 2: Table S2.”
		b)	Report MR estimates of the relationship between exposure and outcome, and the measures of uncertainty from the MR analysis, on an interpretable scale, such as odds ratio or relative risk per SD difference	8-10	“We performed a univariate MR analysis to explore the effect of OSA on … (Figure 2, Additional File 2: Table S3-S4).”<br>“OSA was found to decrease the thickness of temporal pole with global weighted (IVW β (95% CI) =-0.028 (-0.051 to -0.005), P=0.019) … No causal effects of OSA on cognitive performance were found in our study.”<br>“The results of IVW estimate suggested brain cortical structure including thickness of paracentral without global weighted (IVW OR (95% CI) =0.297 (0.116 to 0.760), P= 0.011) …had slightly effects on genetically predicted risk of OSA.”
		c)	If relevant, consider translating estimates of relative risk into absolute risk for a meaningful time period	NA	NA

		d) Consider plots to visualize results (e.g. forest plot, scatterplot of associations between genetic variants and outcome versus between genetic variants and exposure)	8-10	“Figure 2-3”<br>“Additional File 3: Figure S3”
12	**Assessment of assumptions**			
		a) Report the assessment of the validity of the assumptions	8-9	“The details of SNPs used as instrumental variables are displayed in Additional File 2: Table S2. In total, 13 SNPs were extracted to predict OSA genetically and the F statistics for each IV were all greater than the threshold 10 … Cochran Q statistic and funnel plots (Additional File 3: Figure S2, Additional File 2: Table S5). Leave-one-out analysis suggested that the results were not affected by a single outlying variant (Figure 4). All P-values of MR-Egger intercept tests and the MR-PRESSO global tests were greater than 0.05, suggesting no horizontal pleiotropy existed in our MR analysis (Additional File 2: Table S6). Moreover, the results of MR-PRESSO analyses were consistent with IVW method and no outliers were identified (Additional File 2: Table S7).”
		b) Report any additional statistics (e.g., assessments of heterogeneity across genetic variants, such as I^2, Q statistic or E-value)	8-9	“The details of SNPs used as instrumental variables are displayed in Additional File 2: Table S2. In total, 13 SNPs were extracted to predict OSA genetically and the F statistics for each IV were all greater than the threshold 10 … Cochran Q statistic and funnel plots (Additional File 3: Figure S2, Additional File 2: Table S5). Leave-one-out analysis suggested that the results were not affected by a single outlying variant (Figure 4). All P-values of MR-Egger intercept tests and the MR-PRESSO global tests were greater than 0.05, suggesting no horizontal pleiotropy existed in our MR analysis (Additional File 2: Table S6). Moreover, the results of MR-PRESSO analyses were consistent with IVW method and no outliers were identified (Additional File 2: Table S7).”
13	**Sensitivity analyses and additional analyses**			
		a) Report any sensitivity analyses to assess the robustness of the main results to violations of the assumptions	9-10	“In sensitivity analyses, no heterogeneity was observed by Cochran Q statistic and funnel plots (Additional File 3: Figure S2, Additional File 2: Table S5). Leave-one-out analysis suggested that the results were not affected by a single outlying variant (Figure 4). All P-values of

				MR-Egger intercept tests and the MR-PRESSO global tests were greater than 0.05, suggesting no horizontal pleiotropy existed in our MR analysis (Additional File 2: Table S6). Moreover, the results of MR-PRESSO analyses were consistent with IVW method and no outliers were identified (Additional File 2: Table S7).”<br>“Significant heterogeneity was observed in our IVs for the surface area of precuneus with global weighted, surface area of rostral anterior cingulate without global weighted, thickness of paracentral without global weighted, thalamus, general cognitive function and intelligence by Cochran Q statistic and funnel plots (Additional File 3: Figure S4, Additional File 2: Table S10). Heterogeneity was acceptable since we applied the random effects IVW method (54). The MR-Egger intercept did not provided evidence for horizontal pleiotropy (Additional File 2: Table S11). Leave-one-out analysis suggested that the results were not affected by a single outlying variant (Additional File 3: Figure S5). However, MR-PRESSO analysis identified outlier for Thalamus (rs2188399) and Intelligence (rs10119967). The outlier-corrected analyses were consistent with raw test after removing these outlier SNPs (Additional File 2: Table S12).”
	b)	Report results from other sensitivity analyses or additional analyses	9	“Then, we searched 13 SNPs in PhenoScanner V2 and identified 6 SNPs were associated with potential confounders. rs10986730, rs11981973, rs11981973, rs9551988 and rs11981973 were associated with BMI and body fat. rs11075985 was associated with diabetes, vascular or heart problems, sleep duration, snoring, and some obesity-related phenotypes including BMI, body fat, body size, waist circumference, and waist hip ratio. We removed these SNPs and re-conducted MR analysis and found estimates were consistent with the previous result. OSA was still found to decrease the volume of hippocampus (IVW β = 138.87, se = 57.59, P= 0.0159), suggesting that the causal association between OSA on the volume of the hippocampus was not violated by potential confounders (Figure 5).”
	c)	Report any assessment of direction of causal relationship (e.g., bidirectional MR)		
	d)	When relevant, report and compare with estimates from non-MR analyses	11	“Applying LDSC regression, we explored the genetic correlation of OSA with brain structure and cognitive performance, which were shown to have potential causal associations as described above (Figure 6, Additional File 2: Table S14). There was evidence for genetic

				correlations of OSA with general cognitive function (Rg(se)=-0.0913 (0.026), P=5.0e-4) and intelligence (Rg(se)= -0.0907 (0.0242), P=2.0e-4) after Bonferroni correction. However, genetic correlation also suggested OSA was not correlated with brain cortical structure (surface area of precuneus with global weighted, surface area of rostral anterior cingulate without global weighted, thickness of paracentral without global weighted, thickness of superior parietal without global weighted, thickness of temporal pole with and without global weighted), brain subcortical structure (hippocampus and thalamus) and brain structure change covering the lifespan (amygdala and cerebellum white matter).”
		e) Consider additional plots to visualize results (e.g., leave-one-out analyses)	9-10	Leave-one-out analysis suggested that the results were not affected by a single outlying variant (Figure 4).<br>Leave-one-out analysis suggested that the results were not affected by a single outlying variant (Additional File 3: Figure S5).
	**DISCUSSION**			
14	**Key results**	Summarize key results with reference to study objectives	11	“To the best of our knowledge, this is the first large-scale MR study to comprehensively gain the inference about the causal association of OSA with brain structure and cognitive performance. In the present bidirectional MR study, we found that genetically predicted OSA was significantly associated with increased hippocampus volume adjusted for ICV. Nominally causal effects of OSA on brain structures such as thickness of temporal pole with or without global weighted, amygdala structure change and cerebellum white matter change covering lifespan were observed. Bidirectional causal links were also detected between OSA and surface area of precuneus with global weighted, surface area of rostral anterior cingulate without global weighted, thickness of paracentral without global weighted, thickness of superior parietal without global weighted, ICV, thalamus volume, general cognitive function, intelligence and OSA. Our MR findings could provide new insights into the bidirectional links of OSA with brain structural alterations and cognitive function.”
15	**Limitations**	Discuss limitations of the study, taking into account the validity of the IV assumptions, other sources of potential bias, and imprecision. Discuss both direction and magnitude of any potential bias and any efforts to address them	15-16	“A few limitations should be noticed as well. First, since the enrolled patients were mainly Europeans and it’s reported that ethnicity could impact craniofacial anatomy traits and obesity liability in OSA patients, the

				results cannot be generalized to other ancestries (80). Second, the description of brain substructures utilized in our study was based on available neuroimaging GWAS datasets. Since the causal relationship between OSA and hippocampus volume was identified and many observational studies also reported the change in the volume of hippocampus subfields, further MR analysis is expected to estimate the association between OSA and volume of hippocampus subfields if GWAS data is accessed availably ... Besides, for the lack of significant IVs, the genome wide significance threshold was relaxed to P<5e-5 or P<5e-6, which might lead to weak IVs. Since the sample size of GWAS was quite large, this issue was acceptable … Hence, it is conceivable that the outcomes of the two analytical techniques were different.”
16	**Interpretation**			
		a) Meaning: Give a cautious overall interpretation of results in the context of their limitations and in comparison with other studies	11-13	“A number of observation studies have investigated their association applying neuroimaging tools and analytic methods such as MRI, voxel-based morphometry (VBM) and FreeSurfer (55). However, observed phenomena varies substantially across different studies and the findings are not always concordant among different neuroimaging studies, thereby the impacts of OSA on brain subfields are controversial and not yet conclusive (56,57). With regard to hippocampus volume … its pre-processing and different thresholds would markedly influence the results (67,68). Conversely, Freesurfer automated method was proven to be more effective. Besides, these clinical neuroimaging studies were limited by small sample size, different inclusion criteria, lack of OSA-standardized of neuropsychological tests and potential confounders including age, obesity and mixed diseases. The clinical characteristics and experimental conditions varied between studies substantially (69).”
		b) Mechanism: Discuss underlying biological mechanisms that could drive a potential causal relationship between the investigated exposure and the outcome, and whether the gene-environment equivalence assumption is reasonable. Use causal language carefully, clarifying that IV estimates may provide causal effects only under certain assumptions	13	“During OSA, intermittent hypoxia was considered as the key pathological feature and hippocampus is especially vulnerable to hypoxia. It can … Nevertheless, the underlying mechanism of hippocampal volume increase remains unclear and needs further studies to elucidate. And whether OSA can lead to brain function change or neuropsychiatric diseases mediated by the alteration of hippocampus could be expected in the future.”

		c) Clinical relevance: Discuss whether the results have clinical or public policy relevance, and to what extent they inform effect sizes of possible interventions	11-14	"A number of observation studies have investigated their association applying neuroimaging tools and analytic methods such as MRI, voxel-based morphometry (VBM) and FreeSurfer … lack of OSA-standardized of neuropsychological tests and potential confounders including age, obesity and mixed diseases. The clinical characteristics and experimental conditions varied between studies substantially (69)."<br>"Although only one estimate was still significant after the Bonferroni correction, other nominally significant estimates should also be treated carefully. For the thickness of temporal pole … for cerebellum white matter and amygdala throughout the lifespan, which suggested that OSA might affect the process of brain development or aging."<br>"In reverse MR analysis, the increase of thickness of paracentral and superior parietal without global weighted was suggestively associated with a decreased risk of OSA … A recent meta-analysis revealed neurocognitive deficits were evident in children with OSA (79). Hence, whether the cognitive performance could be a marker for OSA required further studies."
17	**Generalizability**	Discuss the generalizability of the study results (a) to other populations, (b) across other exposure periods/timings, and (c) across other levels of exposure	15	"First, since the enrolled patients were mainly Europeans and it's reported that ethnicity could impact craniofacial anatomy traits and obesity liability in OSA patients, the results cannot be generalized to other ancestries (80)."
	**OTHER INFORMATION**			
18	**Funding**	Describe sources of funding and the role of funders in the present study and, if applicable, sources of funding for the databases and original study or studies on which the present study is based	18	"This work was supported by National Natural Science Foundation of China (81571022), Multi-center clinical research project of Shanghai Jiao Tong University School of Medicine (DLY201808)."
19	**Data and data sharing**	Provide the data used to perform all analyses or report where and how the data can be accessed, and reference these sources in the article. Provide the statistical code needed to reproduce the results in the article, or report whether the code is publicly accessible and if so, where	18	"All the data utilized in the present study had been publicly available, and the source of the data had been described in the main text and shown in Additional file 2: Table S1. Code is available from the authors upon request."<br>"We want to acknowledge the participants and investigators of the FinnGen study and ENIGMA Consortium for providing GWAS data."

20	**Conflicts of Interest**	All authors should declare all potential conflicts of interest	18	"The authors declare that they have no competing interests."

This checklist is copyrighted by the Equator Network under the Creative Commons Attribution 3.0 Unported (CC BY 3.0) license.

1. Skrivankova VW, Richmond RC, Woolf BAR, Yarmolinsky J, Davies NM, Swanson SA, et al. Strengthening the Reporting of Observational Studies in Epidemiology using Mendelian Randomization (STROBE-MR) Statement. JAMA. 2021;under review.

2. Skrivankova VW, Richmond RC, Woolf BAR, Davies NM, Swanson SA, VanderWeele TJ, et al. Strengthening the Reporting of Observational Studies in Epidemiology using Mendelian Randomisation (STROBE-MR): Explanation and Elaboration. BMJ. 2021;375:n2233.
