# Supplementary material for: Elucidating the association of obstructive sleep apnea with brain structure and cognitive performance
Source: BMC Psychiatry. 2024 May 6;24:338. doi: 10.1186/s12888-024-05789-x (PMC11071327; doi:10.1186/s12888-024-05789-x)
Supplement: Supplementary file 3 — Supplementary Material 3. [file 12888_2024_5789_MOESM3_ESM.pdf]

Additional file 3: Supplementary Figures.

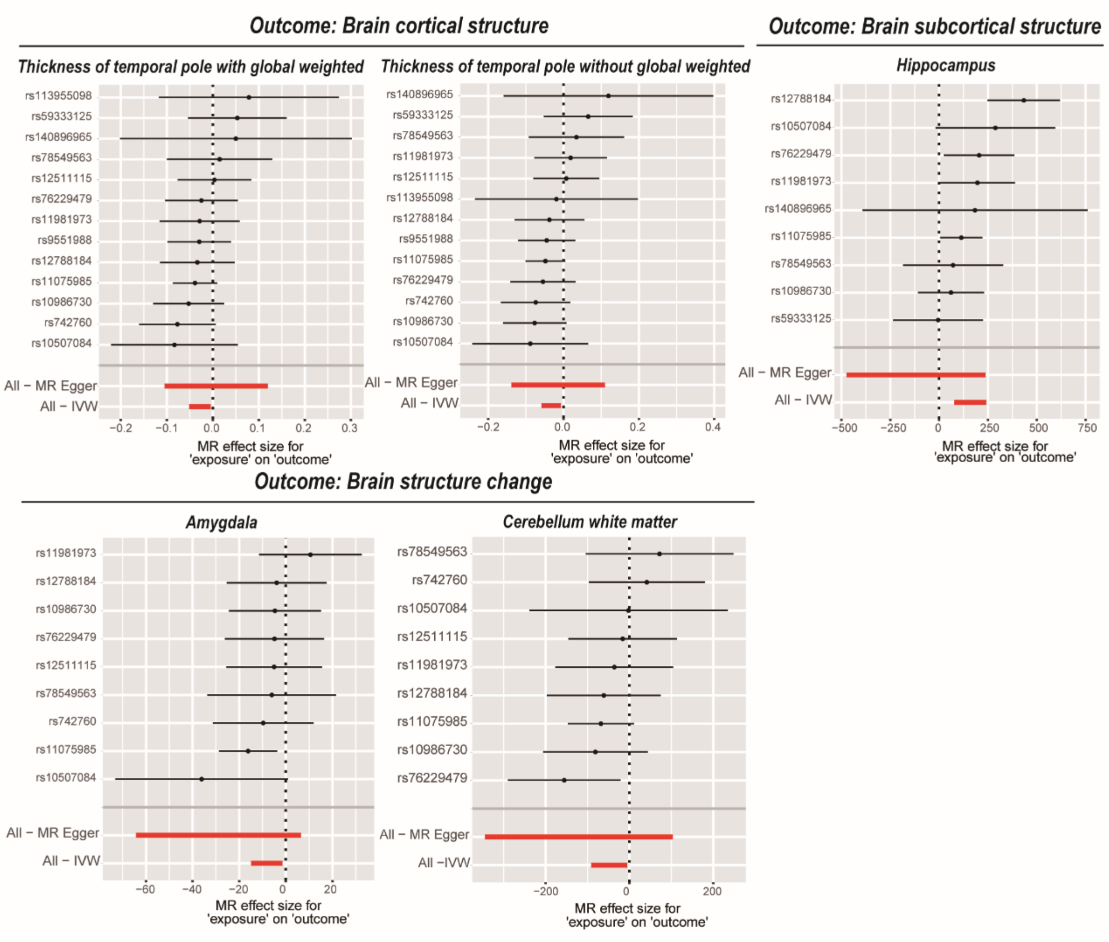

Figure S1. Forest plots show the causal effect of single SNP on brain structure.

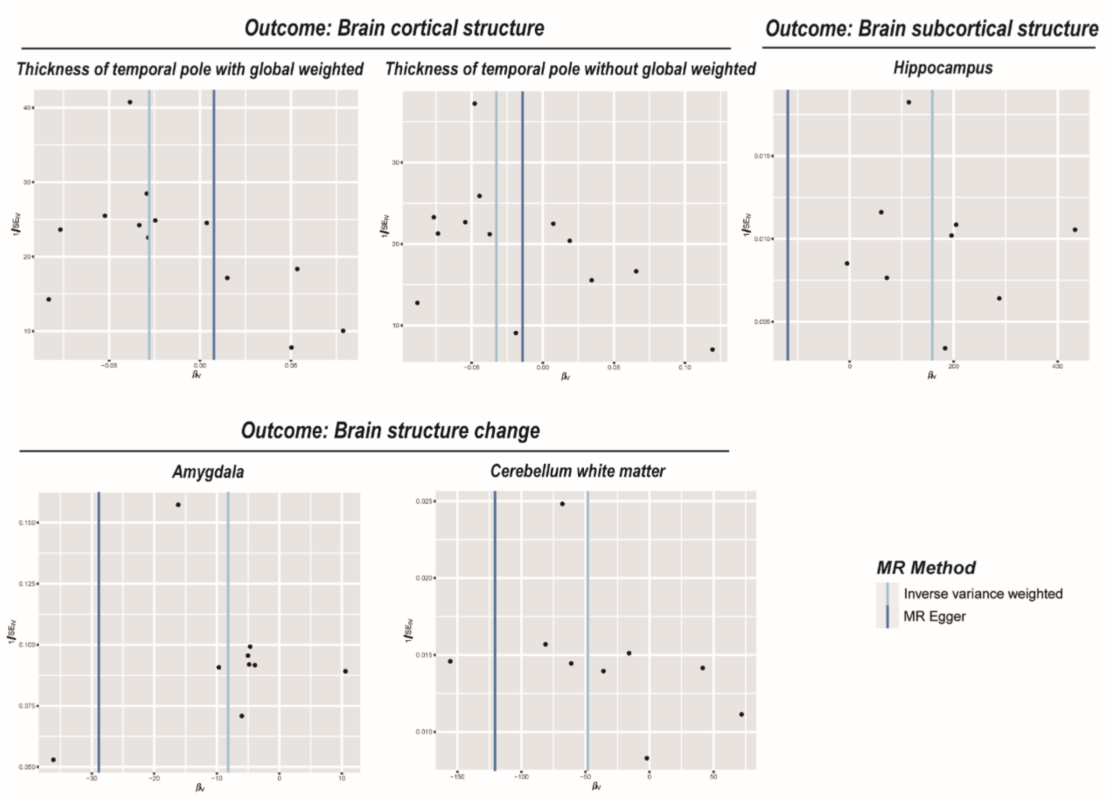

Figure S2. Funnel plots of significant and nominally significant estimates from genetically predicted OSA on brain structure.

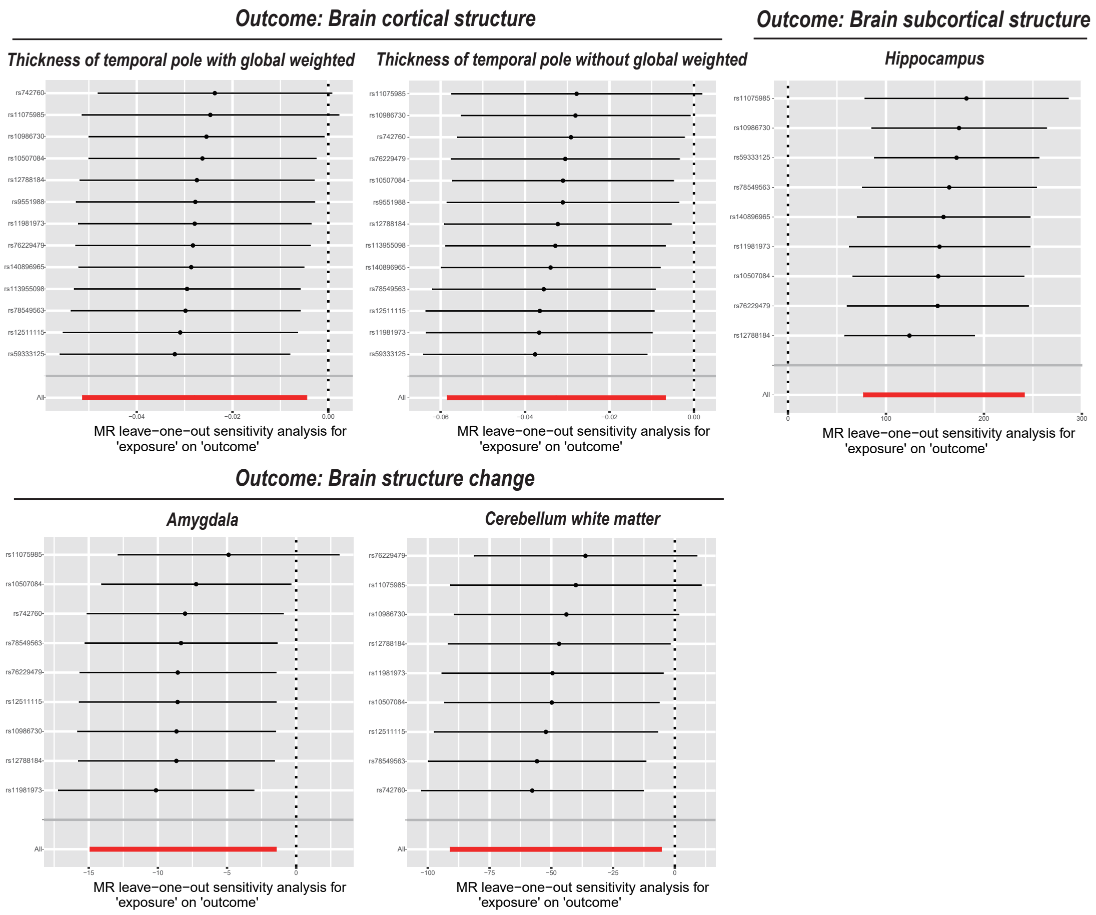

Figure S3. Leave-one-out plots show the causal association of OSA with brain structure and cognitive performance.

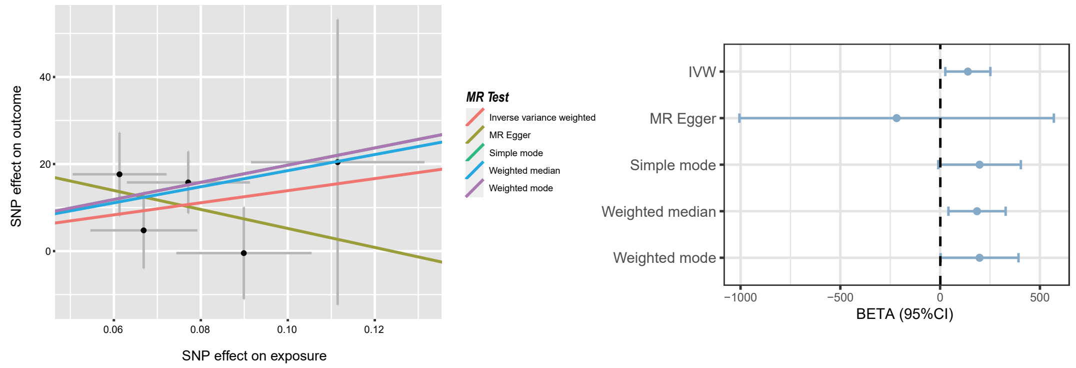

Figure S4. The scatter plot and forest plot show the causal association of OSA with the volume of hippocampus after removing SNPs related to potential confounders.

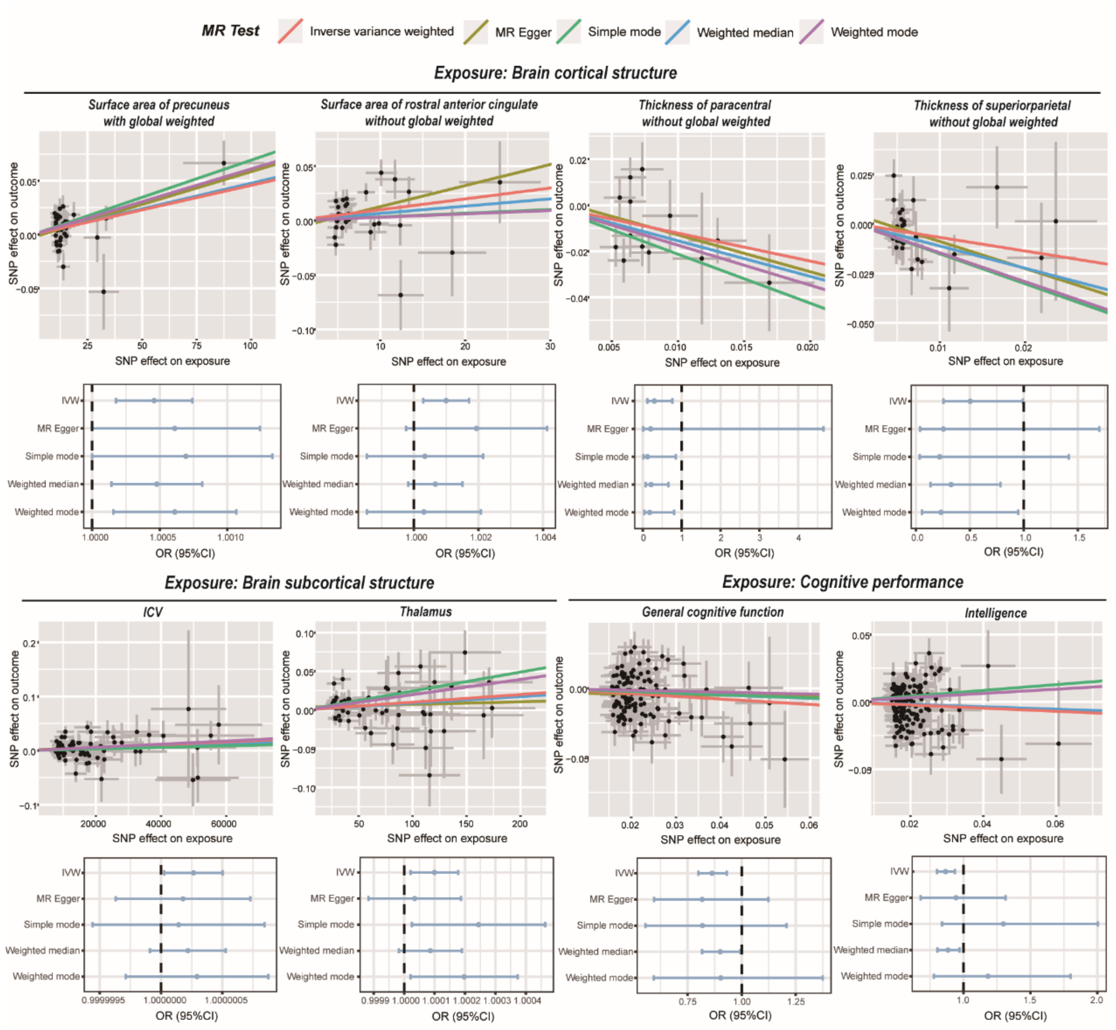

Figure S5. Scatter plots and forest plots show the causal association between neurocognitive features and OSA risk.

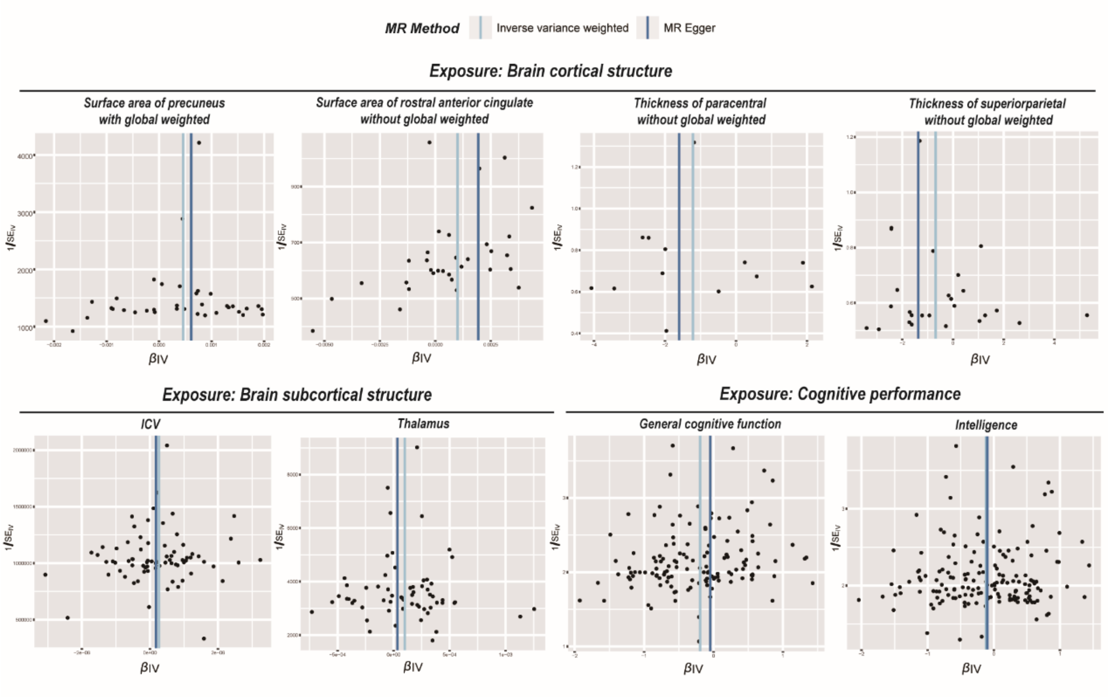

Figure S6. Funnel plots of significant and nominally significant estimates from genetically predicted neurocognitive features on OSA risk.

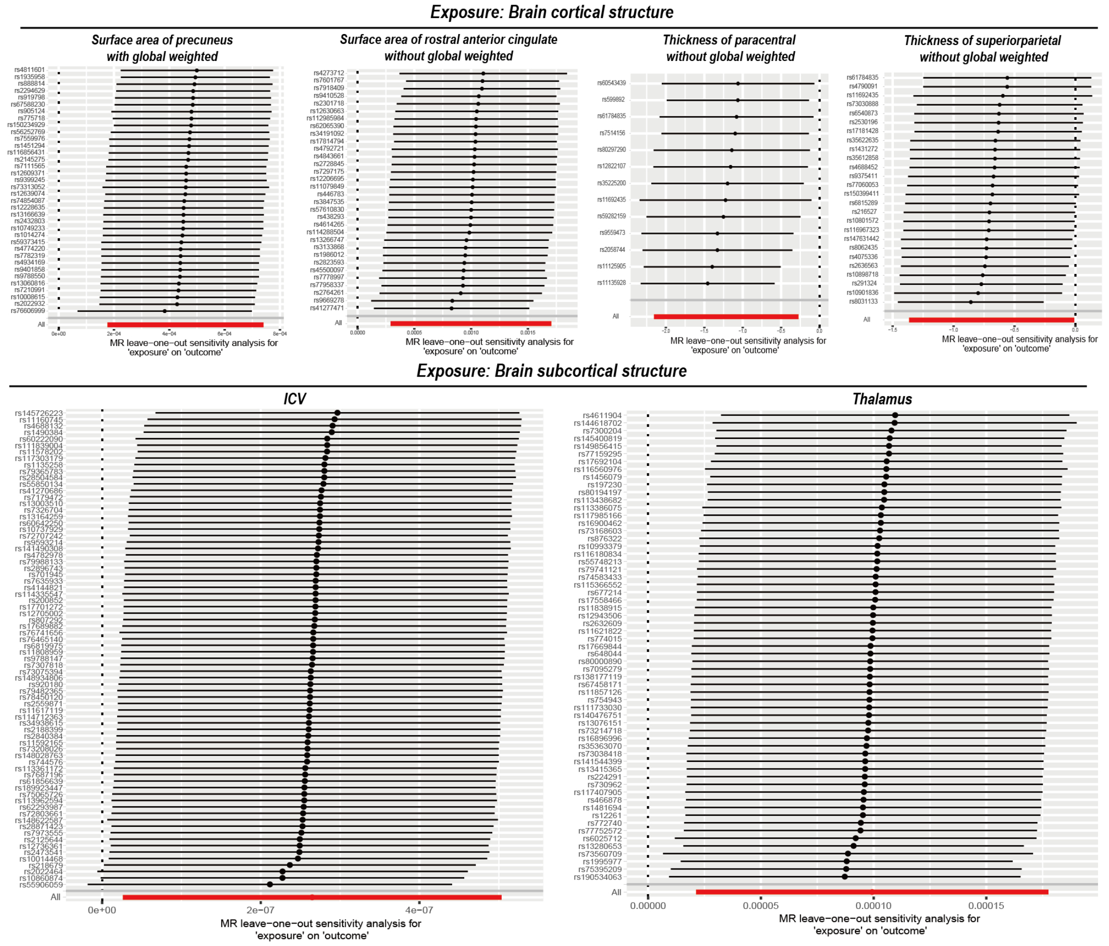

Figure S7. Leave-one-out plots show the causal association between brain structures and OSA risk.

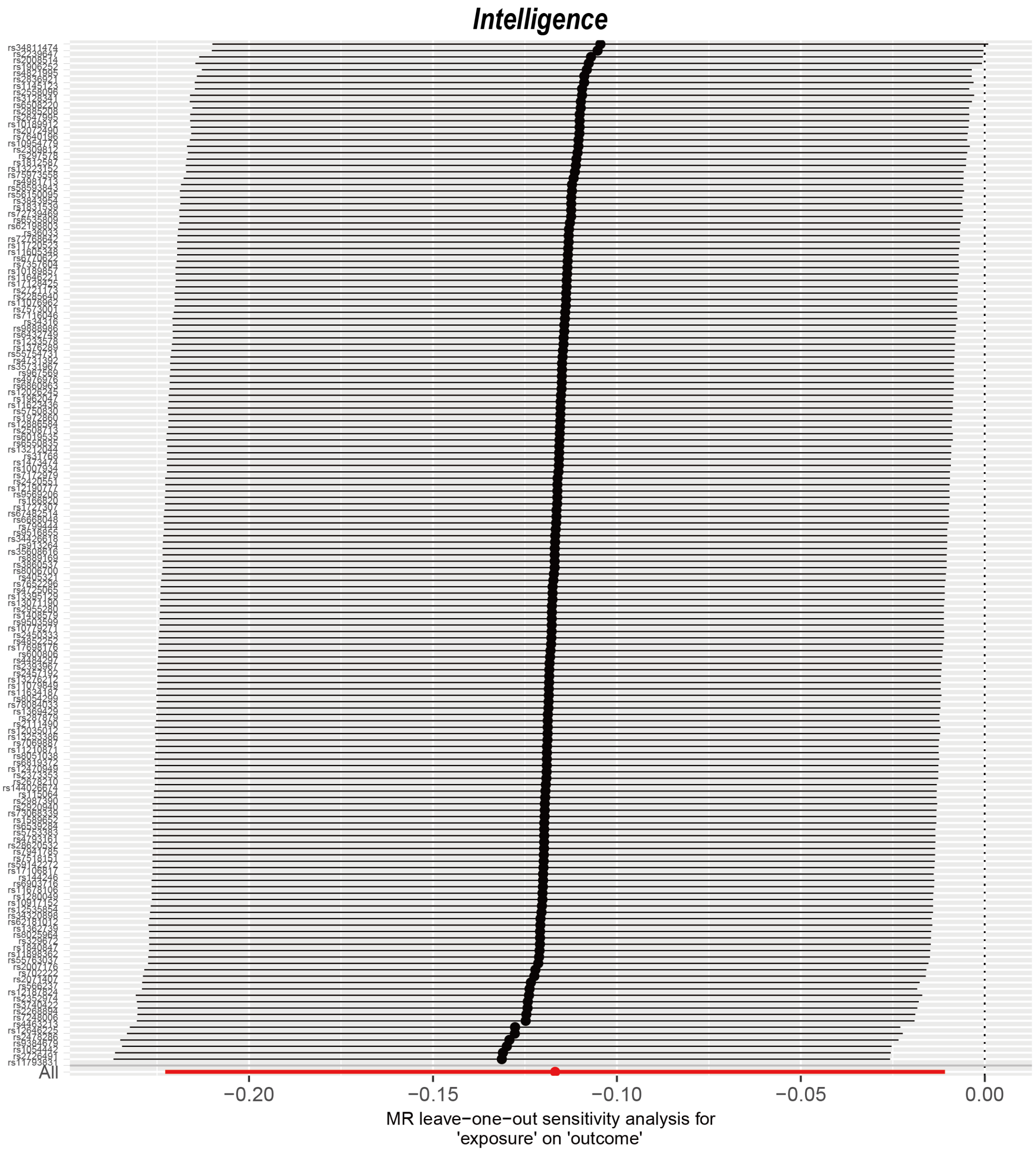

Figure S8. Leave-one-out plots show the causal association between intelligence and OSA risk.

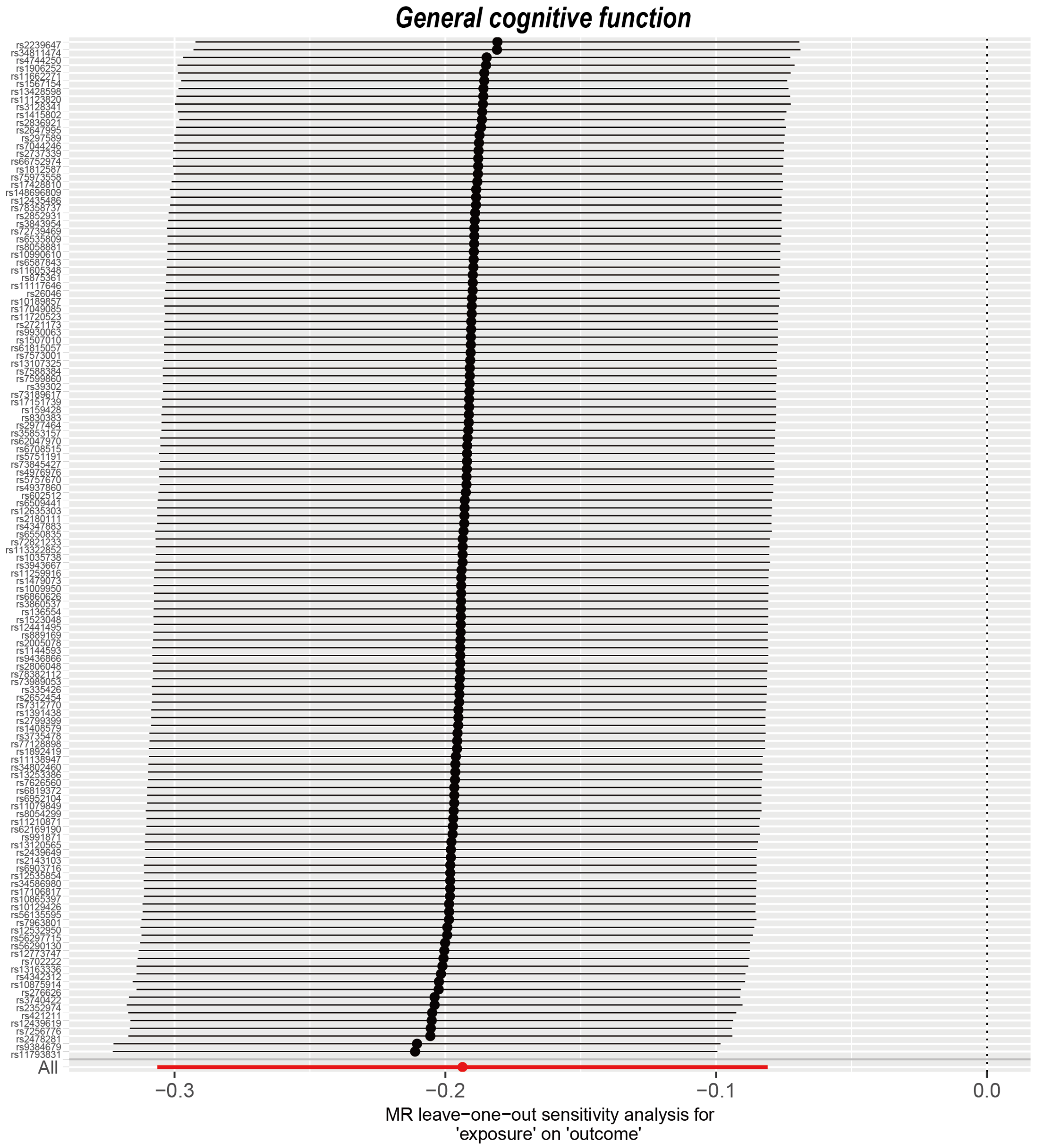

Figure S9. Leave-one-out plots show the causal association between general cognitive function and OSA risk.
